# Framework for advancing rigorous research

**DOI:** 10.7554/eLife.55915

**Published:** 2020-03-04

**Authors:** Walter J Koroshetz, Shannon Behrman, Cynthia J Brame, Janet L Branchaw, Emery N Brown, Erin A Clark, David Dockterman, Jordan J Elm, Pamela L Gay, Katelyn M Green, Sherry Hsi, Michael G Kaplitt, Benedict J Kolber, Alex L Kolodkin, Diane Lipscombe, Malcolm R MacLeod, Caleb C McKinney, Marcus R Munafò, Barbara Oakley, Jeffrey T Olimpo, Nathalie Percie du Sert, Indira M Raman, Ceri Riley, Amy L Shelton, Stephen Miles Uzzo, Devon C Crawford, Shai D Silberberg

**Affiliations:** 1 National Institute of Neurological Disorders and StrokeBethesdaUnited States; 2 iBiologySan FranciscoUnited States; 3 Center for Teaching and Department of Biological Sciences, Vanderbilt UniversityNashvilleUnited States; 4 Department of Kinesiology and Wisconsin Institute for Science Education and Community Engagement, University of Wisconsin - MadisonMadisonUnited States; 5 Department of Anesthesia, Critical Care and Pain Medicine, Massachusetts General HospitalBostonUnited States; 6 Department of Brain and Cognitive Science, Institute of Medical Engineering and Sciences, the Picower Institute for Learning and Memory, and the Institute for Data Systems and Society, Massachusetts Institute of TechnologyBostonUnited States; 7 Department of Biology and Program in Neuroscience, Brandeis UniversityWalthamUnited States; 8 Harvard Graduate School of Education, Harvard UniversityCambridgeUnited States; 9 Department of Public Health Sciences, Medical University of South CarolinaCharlestonUnited States; 10 Planetary Science InstituteTucsonUnited States; 11 Cellular and Molecular Biology Graduate Program, University of MichiganAnn ArborUnited States; 12 The Concord ConsortiumEmeryvilleUnited States; 13 Department of Neurological Surgery, Weill Cornell Medical CollegeNew YorkUnited States; 14 Department of Biological Sciences, Duquesne UniversityPittsburghUnited States; 15 Solomon H. Snyder Department of Neuroscience, Johns Hopkins School of MedicineBaltimoreUnited States; 16 Carney Institute for Brain Science, Department of Neuroscience, Brown UniversityProvidenceUnited States; 17 Centre for Clinical Brain Sciences, University of EdinburghEdinburghUnited Kingdom; 18 Biomedical Graduate Education, Georgetown University Medical CenterWashingtonUnited States; 19 MRC Integrative Epidemiology Unit, School of Psychological Science, University of BristolBristolUnited Kingdom; 20 Oakland UniversityRochesterUnited States; 21 Department of Biological Sciences, The University of Texas at El PasoEl PasoUnited States; 22 National Centre for the Replacement, Refinement and Reduction of Animals in Research (NC3Rs)LondonUnited Kingdom; 23 Department of Neurobiology, Northwestern UniversityEvanstonUnited States; 24 ComplexlyMissoulaUnited States; 25 Center for Talented Youth and School of Education, Johns Hopkins UniversityBaltimoreUnited States; 26 New York Hall of ScienceFlushing Meadows Corona ParkUnited States

**Keywords:** research culture, scientific rigor, education, mentoring, None

## Abstract

There is a pressing need to increase the rigor of research in the life and biomedical sciences. To address this issue, we propose that communities of 'rigor champions' be established to campaign for reforms of the research culture that has led to shortcomings in rigor. These communities of rigor champions would also assist in the development and adoption of a comprehensive educational platform that would teach the principles of rigorous science to researchers at all career stages.

The scientific enterprise relies on mentors teaching their students and trainees how to design and conduct studies that produce reliable scientific knowledge. A crucial part of this is teaching students and trainees how to minimize the risks that chance observations, subconscious biases, or other factors might lead to incorrect or inflated claims. However, as the demands on mentors increase, some of them unintentionally overlook this crucial aspect of scientific investigation, meaning that students and trainees are not taught how to distinguish between high- and low-quality evidence when working on their own studies and when reading about other studies (Ioannidis et al., 2014; Bosch and Casadevall, 2017; Landis et al., 2012).

Additional complications stem from the welcome rise in team-based science and a greater sophistication and range of experimental techniques (National Research Council, 2015), which may, in part, be driven by a feeling that only exciting and complete stories will appeal to journals and funders (Nosek et al., 2012; Casadevall et al., 2016). Increasingly, an individual scientist cannot be an expert in all the techniques used in a research project.

Taken together, these developments suggest that enhanced training in the fundamental principles of rigorous research common to most, if not all, experimental practices is needed to ensure that the outputs of scientific research remain reliable and robust. Such principles include strong reasoning and inference based on valid assertions, which requires the proper interpretation of uncertainty and a motivation to identify inconsistencies (Bosch and Casadevall, 2017; Casadevall and Fang, 2016; Munafò and Davey Smith, 2018; Wasserstein et al., 2019). For studies that test hypotheses, researchers should: clearly define interventions; identify and disclose possible confounding factors; transparently report project workflows, experimental plans, methods, data analyses, and any divergence from pre-planned procedures; and fully report their competing interests (see https://www.equator-network.org/ for reporting guidelines). The requirements for studies intended to generate hypotheses will be different but should be equally described (Dirnagl, 2019).

Before formulating solutions to these issues, we assessed current training practices at the graduate and postdoctoral levels by surveying all 41 institutions in the United States that held at least one training grant from the National Institute of Neurological Disorders and Stroke (NINDS) in May 2018. Only 5 of the 37 institutions that responded to the survey reported providing a course predominantly dedicated to principles of rigorous research, with others using a range of approaches – such as seminars, lectures within other coursework, workshops, and informal mentoring – to teach good research practices. However, few if any of the institutions covered the full range of principles that need to be learned and understood. Although the sample in our survey was small, the responses reinforced the common belief that formal training in rigorous research needs to be enhanced (Ioannidis et al., 2014; Munafò et al., 2017).

While numerous training materials related to rigorous research are available online, finding suitable materials and assembling them into a cohesive course is challenging. Having access to a free, organized suite of educational resources could greatly reduce the energy barrier for institutions and scientists to implement enhanced training at all levels, from undergraduate education to faculty professional development.

Towards this end NINDS convened a workshop attended by a range of stakeholders: basic, translational, and clinical neuroscientists; scholars of education and science communication; educational platform developers; and trainees. Although neuroscience served as a focal point, the four outcomes of the discussions apply widely across the biomedical sciences: i) there is a clear need for a platform that teaches the principles of rigorous research and covers the needs of scientists at all career stages; ii) effective educational interventions should lead to measurable behavioral change; iii) academic institutions need to play a proactive role in promoting rigorous research practices; iv) progress in this area will require cultural change at academic institutions, funders, and publishers (Casadevall et al., 2016; Munafò et al., 2017; Collins and Tabak, 2014; Begley et al., 2015; Casadevall and Fang, 2012).

## Building communities of rigor champions

To unleash the motivation for a cultural change evident in discussions between the authors and early-career researchers and others, and to provide momentum for change across different sectors, we propose the establishment of inter- and intra-institutional communities of 'rigor champions' who are committed to promoting rigor and transparency in research. We know there are many such individuals working at different levels of seniority in different types of organizations (such as universities, funders, publishers, and scientific societies), but they often feel isolated and under-resourced. To seed this effort and to help like-minded individuals in different organizations to find each other and join forces, NINDS has created a website for researchers, educators, trainees, organizational leaders and others who are passionate about the issues discussed here. This website includes currently available resources for making science more rigorous and transparently reporting results, as well as instructions for identifying yourself as a rigor champion.

More information about the different activities that these communities could undertake are given in Table 1. Researchers, educators and trainees are best placed to collaborate on new tools, share best practices, and promote rigorous research in their local scientific communities. Societies are in a position to advocate for widespread policy changes, while funders and journals have important gatekeeping roles (Collins and Tabak, 2014; McNutt, 2014; Cressey, 2015; PLOS Biology, 2018). The recently established UK Reproducibility Network (Munafò et al., 2020) and the PREMIER project (Dirnagl et al., 2018), both of which aim to improve scientific practices, may serve as models for these communities.

**Table 1.** Activities for communities of rigor champions to promote the principles of rigorous research.

Community	Intra-organizational activities	Inter-organizational activities
*Trainees*	• Promote transparency and other rigorous practices among colleagues and mentors • Advocate for resources to facilitate rigorous research practices	• Share institutional resources and practices in education and training • Call for changes in institutional culture and policies
*Researchers*	• Transparently report all experiments, including neutral outcomes • Promote rigorous practices among colleagues and trainees • Call for changes to institutional culture, policies, and infrastructure	• Share effective training practices and useful laboratory resources • Coordinate with the broader scientific community to promote better incentive structures
*Educators*	• Suggest improvements to available resources that address rigor • Integrate rigorous research principles into all coursework	• Share resources and educational best practices • Share effective learning evaluation methods
*Institutional Leaders*	• Enact policies and support infrastructure to incentivize transparency and other rigorous research practices • Explicitly incorporate mentoring, collaboration, and rigorous research practices into promotion procedures • Initiate and share outcomes from piloted educational resources	• Support and promote communities of rigor champions • Disseminate policy changes, new initiatives, educational successes, and implementation strategies • Develop tangible outcome measures to evaluate impact
*Journal Editors and Reviewers*	• Promote thorough review of research practices in publications • Explicitly support research transparency and neutral outcomes • Educate reviewers on which scientific practices are valued by the journal	• Collaborate to implement best practices consistently across different publishers
*Scientific Societies and Organizations*	• Support the founding of communities of rigor champions • Compile and encourage best practices used by the scientific community • Host workshops and educational materials for members	• Promote and maintain communities of rigor champions • Encourage institutional policies that promote research quality and effective education
*Funding Organizations*	• Emphasize attention to rigor in peer review • Reward rigorous research practices and outstanding mentorship • Support infrastructure for transparent and rigorous science • Support educational resources and initiatives	• Support and promote communities of rigor champions • Share best practices for incentivizing rigorous research and educating scientists • Develop partnerships to support better training and facilitate cultural changes

NINDS, for example, has proactively sought effective approaches to support greater transparency in reporting. An NINDS meeting with publishers led to changes in journal policies regarding transparency of reporting at various journals (Nature, 2013; Kelner, 2013). Recommendations for greater transparency at scientific meetings stemmed from an NINDS roundtable with conference organizing bodies (Silberberg et al., 2017) and are being piloted by the Federation of American Societies for Experimental Biology (FASEB). To recognize outstanding mentors, NINDS established the Landis Mentoring Award, and by providing greater stability to meritorious scientists though the NINDS R35 Program, it is anticipated that the pressures to rush studies to publication will be mitigated.

In particular we hope that leaders at academic institutions – such as department chairs, deans, and vice-presidents of research – will become involved because they are uniquely placed to shape the culture and social norms of institutions (Begley et al., 2015). For example, faculty evaluation criteria should be modified to place greater emphasis on data sharing, methods transparency, demonstrated rigor, collaboration, and mentoring, with less emphasis on the number of publications and journal impact factors (Casadevall and Fang, 2012; Moher et al., 2018; Bertuzzi and Jamaleddine, 2016; Lundwall, 2019; Strech et al., 2020; Casci and Adams, 2020; see also https://sfdora.org/read). When publications are being evaluated, rigorously obtained null results should be valued as highly as positive findings. Institutional leaders are also uniquely placed to ensure that scientific rigor is properly taught to trainees and incorporated into day-to-day lab work (Casadevall et al., 2016; Begley et al., 2015; Bosch, 2018; Button et al., 2020). Moreover, evaluations of trainees should emphasize experimental and analytic skills rather than where papers are published.

## Building an educational resource for rigorous research

**Figure 1.**
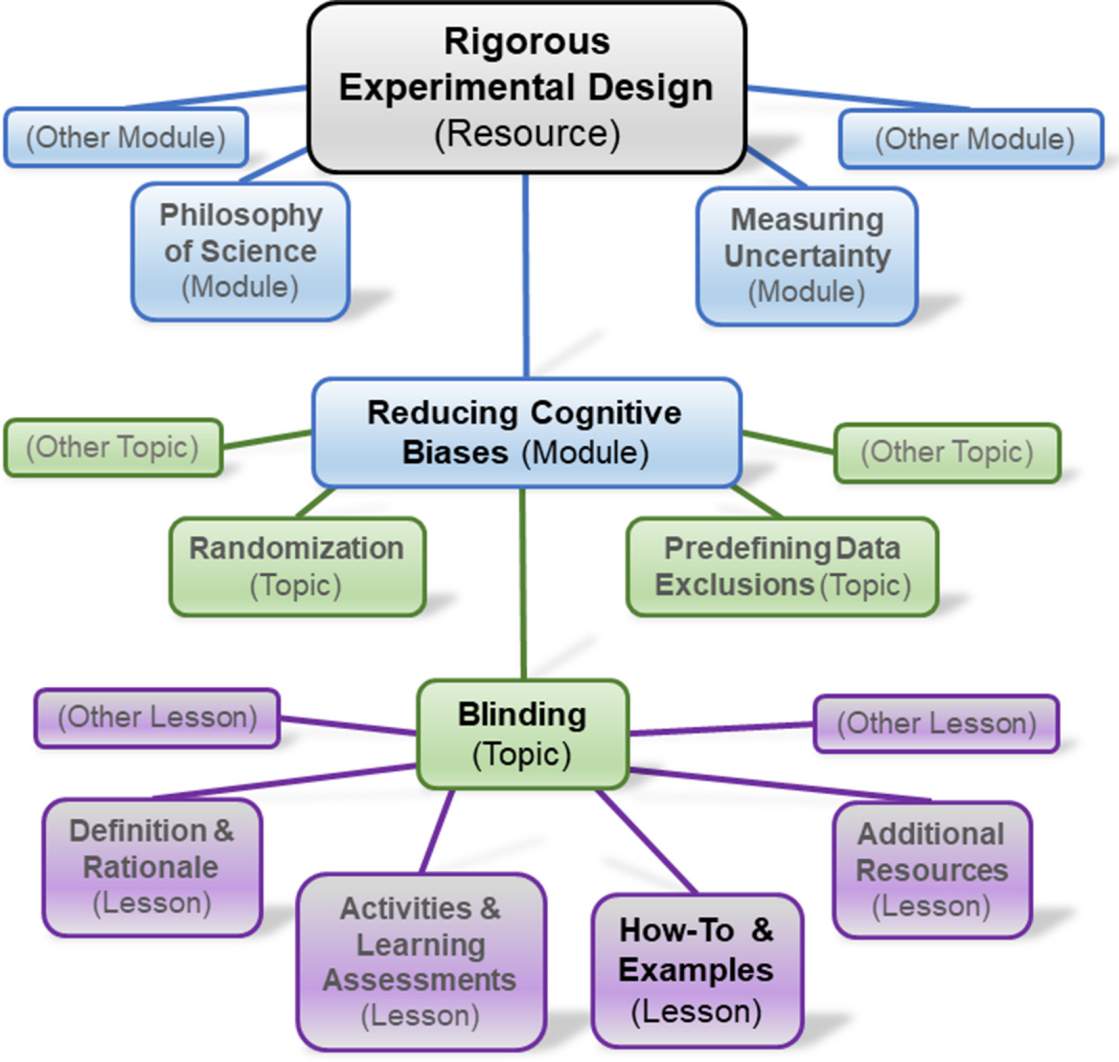
Outline of an educational resource on the principles of rigorous research suitable for a variety of audiences. We envision a comprehensive resource that can be used by scientists at all stages of their career to explore the principles of rigorous research at various levels of detail. We envision modules on a range of topics (such as reducing cognitive biases), each of which contains a number of topics (such as blinding), each of which contains a number of lessons (such as practical examples).

The establishment of communities of rigor champions will set the stage for the creation of an educational platform designed by the scientific community to communicate the principles of rigorous research. Given the rapid evolution of technologies and learning practices, it is difficult to predict what resource formats will be most effective in the future, so the platform will need to be open and freely available, easily discoverable, engaging, modular, adaptable, and upgradable. It will also need to be available during coursework and beyond so that scientists can use it to answer questions when they are doing research or as part of life-long learning (Figure 1). This means that the platform will have to embody a number of principles of effective teaching and mentoring (see Table 2).

**Table 2.** Key elements of teaching and learning to include in an educational resource on the principles of rigorous research.

Key element	Teaching and learning principle
*Clear learning objectives*	Define the learning objectives upfront, identify ways to measure achievement of these objectives, and then design activities to support learning (Bradforth et al., 2015).
*Inquiry-based learning*	Encourage students to pose their own questions, apply commonly used tools and methods to actively explore their questions, and provide evidence when explaining phenomena (Bradforth et al., 2015; Corwin et al., 2015; Minner et al., 2010; Handelsman et al., 2004).
*Relevance*	Provide feedback on real-world experiments, whether in the classroom or the laboratory, as a way to demonstrate relevance and stimulate interest. Opportunities for personalized application and discussion in the local setting with the help of a facilitator’s guide are particularly critical, as adults typically learn most effectively when given the opportunity for immediate personal utility and value (Walkington and Bernacki, 2018). Emphasize the ability to contribute to a larger purpose or gain social standing (Yeager et al., 2014).
*Individuality*	Include a range of approaches to teaching and learning to accommodate different levels of knowledge and skills, motivations, and senses of self-efficacy (Walkington and Bernacki, 2018; Raman, 2014).
*Self-efficacy*	Allow individuals to gain self-efficacy by experiencing a feeling of progress, being challenged in low-stakes environments, and working through confusing concepts successfully (D’Mello et al., 2014). This is more effective when the person feels psychologically safe to take risks and fail in front of their local scientific community.
*Belonging*	Facilitate learning, foster collaboration, and recognize diverse perspectives in order to encourage learners to gain agency and forge a connection with the intellectual community (Bjork et al., 2013; Brown and Adler, 2008).
*Recognition of complexity*	Include complexity and inconsistencies in training examples rather than simplification for the sake of a persuasive story (Howitt and Wilson, 2014; Coleman, 1987). This counteracts the drive to smooth over inconvenient but potentially important details and highlights the importance of confounding variables, potential artefactual influences, reproducibility, and robustness of the findings.
*Cultivation of growth*	Nurture positive behaviors, like acknowledging and learning from mistakes, rather than penalize imperfect practices (Alberts et al., 2015). Mentors at all career stages are encouraged to model these positive behaviors and to share their own failures, the drudgery and frustrations of science, and their approaches to coping emotionally and growing intellectually while maintaining rigorous research practices.
*Assessment of behavioral change*	Measure success via gains in learner competency and changes to their real-world approaches to research. Changes in laboratory practice could be assessed by user self-reports, by analysis of research presented at meetings (Silberberg et al., 2017) and in publications (MacLeod et al., 2015), or by querying scientists on whether discussions with their mentors and colleagues led to changes in laboratory and institutional culture. Collaborate from the beginning with individuals who specialize in assessment design in higher education settings (Bradforth et al., 2015).

We envision the platform being developed via a hub-and-spoke approach as discussed at a recent National Advisory Neurological Disorders and Stroke Council meeting. A centralized mechanism (the 'hub') will provide financial and infrastructural support and guidance (possibly via a steering committee) and facilitate sharing and coordination between groups, while rigor champions will come together to design specific modules (spokes) for the platform by using existing resources or designing new ones from scratch as needed. We envision worldwide teams of experts collaborating on building and testing the resource. Rigor champions with experience in defining clear learning objectives, building curricula, and evaluating success, for example, will collaborate with content experts to design topics needed in the resource. Importantly, potential users will be involved from the beginning of the development stage, and onwards through the design and implementation stages, to provide feedback about effectiveness and usability.

Given the importance of being able to measure the effectiveness (or otherwise) of the platform (Table 2), individual components should be released publicly as they are completed to allow educators and users to iteratively test and improve the resource as it unfolds. As with science itself, the developers will need to experiment with content and delivery. If the resource does not improve the comprehension and research practice of individuals, or add value to the research community, rigorous approaches should be applied to improve it.

Once a functioning and effective resource has been built, it will be essential to promote its use and adoption. One approach would be to host 'train-the-trainer' programs (Spencer et al., 2018; Pfund et al., 2006): those involved in building the resource share it with small groups of mentors, who are then better equipped to use the resource with their own mentees and to encourage their colleagues to use it. This form of dissemination also creates buy-in from mentors who need to model the behaviors they are teaching. Rigor champions, meanwhile, can encourage their institutions and colleagues to adopt and use the resource.

Setting up and supporting communities of rigor champions and developing educational resources on rigorous research will be complex and likely require multiple sources of support. However, with the participation of all sectors of the scientific enterprise, the actions proposed herein should, within a decade, lead to improvements in the culture of science as well as improvements in the design, conduct, analysis, and reporting of biomedical research. The result will be a healthier and more effective scientific community.

### Disclaimer

The content of this publication does not necessarily reflect the views or policies of the Department of Health and Human Services, nor does mention of trade names, commercial products, or organizations imply endorsement by the US Government.
